# T-Bar Utilization for Concomitant Coronary Artery Bypass Graft Operation and Left Upper Lobectomy

**DOI:** 10.1155/2016/8760849

**Published:** 2016-03-02

**Authors:** Fotios Mitropoulos, Meletios A. Kanakis, Anastasios Apostolou, Andrew Chatzis, Constantinos Contrafouris, Christos Apostolidis, Achilleas Lioulias

**Affiliations:** ^1^Department of Pediatric and Congenital Heart Surgery, Onassis Cardiac Surgery Center, Kallithea, 17674 Athens, Greece; ^2^Department of Thoracic Surgery, Sismanoglio General Hospital, Marousi, 15126 Athens, Greece

## Abstract

Management in patients with coexisting coronary artery disease and lung carcinoma is usually a two-stage operation, with the cardiac surgery procedure followed by pulmonary resection at a later time. Delayed tumor resection on the other hand may be detrimental. Off-pump coronary artery bypass grafting could facilitate concomitant lung resection at one stage via median sternotomy. T-bar retractor may be a useful tool in the surgical approach of this combined operation.

## 1. Introduction

Traditionally, management in patients with coexisting coronary artery disease (CAD) and lung carcinoma is a two-stage operation, with the cardiac surgery procedure followed by pulmonary resection at a later time. This is because the systemic heparinization and coagulation disorders make the lung resection operation more difficult.

Delayed tumor resection on the other hand may increase the possibility of metastasis; the immunosuppressive effects of CBP may increase the tumor growth and dissemination. With the development of coronary artery surgery, especially off-pump coronary artery bypass grafting (OPCABG), many surgeons prefer to perform cardiac surgery and pulmonary resection simultaneously. Avoidance of extracorporeal circulation may decrease the incidence of postoperative complications and saves the patient from a second procedure, which includes another general anesthetic and incision [1, 2].

## 2. Case Report

**Figure 1:**
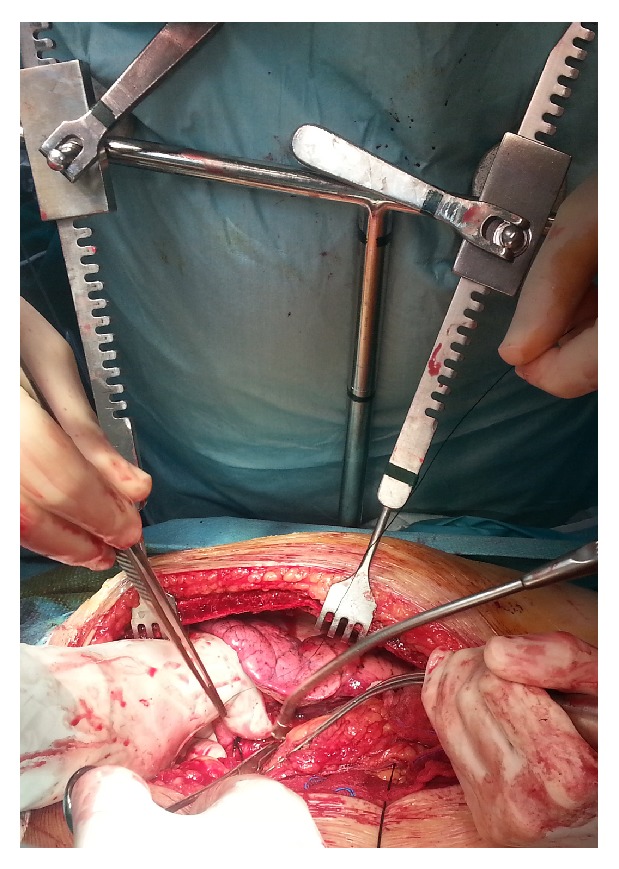
Intraoperative photo depicting the utility of T-bar in accessing left upper lobe.

An 80-year-old man was admitted with diagnosis of non-small-cell lung carcinoma of the left upper lobe. During the preoperative work-up, ECG changes suggested further investigation. Finally, cardiac catheterization showed occlusion of left anterior descending coronary artery 90% proximally. Ejection fraction was 45%. Concomitant coronary artery bypass grafting (CABG) and lung resection were decided. Following general anesthesia, single lumen endotracheal intubation was used. After median sternotomy the left internal thoracic artery was harvested by using the T-bar retractor. Using heart stabilizer and *β* blockade, LAD was opened and LIMA was anastomosed to it. At this point we used again the Τ-bar for retracting the left hemithorax which gave us very good exposure and access to left upper lobe (Figure 1). LIMA was protected with carbasus from the operating field and lobectomy was carried out without complications. Lymph node dissection of stations 5, 6, 7, 8, 9, 10, and 11 was also performed. At the end two chest thoracic drain tubes were used, one in the left hemithorax and the other in the mediastinum, and the incision was closed in continuous layers. The patient was extubated in the next day maintaining good haemodynamic status in the ICU and he was transferred to the ward. Postoperative course was uneventful and he was discharged on the 6th postoperative day. Pathology examination showed primary adenocarcinoma of the lung T1N0M0. At 18-month follow-up period, the patient is in an excellent clinical status with no evidence of cancer disease.

## 3. Discussion

There are two strategies for patients with coexisting coronary artery disease and lung cancer: a staged approach with revascularisation preceded by 2-3 weeks of lung resection or a single combined procedure of coronary artery bypass surgery and lung resection.

The advantages of staged procedure are stable revascularised myocardium at the time of lung resection; there is no issue of bleeding, lateral thoracotomy with full node access. Disadvantages are delay of compelling lung surgery, two thoracotomies in quick succession, and the use of anticoagulants after CABG. Also, there is evidence that the revascularisation procedure compromises the immune system and therefore the risk of tumor spread is not negligible. The advantages of synchronous treatment are that the patient undergoes one operation and there is no delay between the CABG and resection for lung carcinoma. Disadvantages are the chance of unstable myocardium during lung resection and potential bleeding related to heparinization especially with CBP; left lower lobectomy is difficult with median sternotomy.

Some surgeons suggest that only stage I, and maybe limited stage II disease, which does not show other features of aggressive disease (poorly differentiated, large cell undifferentiated, neuroendocrine, multiple node stations, requiring pneumonectomy, high SUV on PET, and bulky disease) should be considered for a combined approach [3]. Left posterolateral thoracotomy is considered ideal for lung resection and provides excellent exposure of the circumflex artery and left anterior descending artery [1]. However, several reports suggest successful combined pulmonary resection and CABG via median sternotomy [3, 4]. We have chosen the latter method as this particular patient had compromised cardiac function and median sternotomy offered a safer approach for potential CPB institution.

Using the T-bar for concomitant off-pump CABG and left upper lobectomy via median sternotomy proved valuable in treating our patient. The instrument offered satisfactory access to left lung allowing safe dissection of the pulmonary artery and vein branches and provided adequate exposure for lymph node dissection. Even the area of the pulmonary ligament as also the left lower lobe could be safely approached with appropriate adjustments of the T-bar.

## 4. Conclusion

In this case report, the utility of T-bar rises to prominence as a valuable tool for easier approach of the left lung via median sternotomy.

## References

[1] Ahmed A. A. M., Sarsam M. A. I. (2001). Off-pump combined coronary artery bypass grafting and left upper lobectomy through left posterolateral thoracotomy. *Annals of Thoracic Surgery*.

[2] Zhang Y., Wu Y., Yuan B. (2010). Coronary artery bypass grafting with concomitant resection for carcinoma of lung. *Journal of Biomedical Research*.

[3] Danton M. H. D., Anikin V. A., McManus K. G., McGuigan J. A., Campalani G. (1998). Simultaneous cardiac surgery with pulmonary resection: presentation of series and review of literature. *European Journal of Cardio-Thoracic Surgery*.

[4] Saxena P., Tam R. K. W. (2004). Combined off-pump coronary artery bypass surgery and pulmonary resection. *Annals of Thoracic Surgery*.

